# A surgical challenge: correcting hallux duplication and syndactyly in a pediatric patient with Gaucher disease and unprecedented skeletal manifestations

**DOI:** 10.1093/jscr/rjaf587

**Published:** 2025-08-06

**Authors:** José F Contreras, Carlos Diaz Q

**Affiliations:** Research Medical Department, Panamerican University, 27 Avenida, Finca El Naranjo, 8-36, Zona 4, Mixco, Guatemala City 01057, Guatemala; Research Medical Department, Universidad Francisco Marroquín, 13 av, Guatemala City 01011, Guatemala

**Keywords:** Gaucher disease, hallux duplication, syndactyly, pediatric surgery, osteoporotic bone

## Abstract

We present a rare case of a pediatric patient diagnosed with Gaucher disease, a lysosomal storage disorder, co-occurring with hallux duplication and syndactyly, congenital foot malformations. The patient exhibited extreme and uncommon manifestations of Gaucher disease, including massive splenomegaly and severe osteoporosis localized to the affected extremities, which complicated the treatment of the foot deformities. This case is notable due to the unique interaction between two genetically distinct conditions. We explore the clinical implications, treatment challenges, and potential genetic factors that may underlie the co-existence of these rare disorders. This case highlights the importance of comprehensive diagnosis and careful management when dealing with complex, overlapping conditions.

## Introduction

Gaucher disease (GD) is one of the most common lysosomal storage disorders, caused by a deficiency of the enzyme glucocerebrosidase. This results in the accumulation of glucocerebroside in various organs, particularly in the spleen, liver, bone marrow, and sometimes the lungs and central nervous system. The clinical manifestations of Gaucher disease can range from mild to severe and typically include splenomegaly, hepatomegaly, bone pain, osteoporosis, and neurological impairment [1].

The disease is categorized into three types: Type 1 (non-neuropathic), Type 2 (acute neuronopathic), and Type 3 (chronic neuronopathic), with Type 1 being the most common and typically affecting the liver, spleen, and bone marrow without significant neurological involvement [2]. Across all GD types, incidence estimates ranged 0.45–25.0/100 000 live births [3]. The incidence of hallux duplication is 2.4/100 000 in South America [4].

Hallux duplication and syndactyly are rare congenital malformations that often require surgical correction to improve function and appearance. This case highlights the complex surgical challenges posed by their coexistence with Gaucher disease. The patient's fragile bone structure demanded a meticulous approach to minimize intraoperative fracture risks, raising important considerations for managing concurrent skeletal disorders and congenital malformations.

## Case presentation

A 6-year-old female presented with congenital hallux duplication and syndactyly of the left foot, causing functional limitations, gait abnormalities, and esthetic concerns. The family history was unremarkable, and the birth history was normal. Clinical evaluation revealed foot swelling, hallux duplication, and syndactyly of adjacent toes. The patient also exhibited worsening bone pain, poor growth, delayed motor milestones, and fatigue. Laboratory results showed anemia (hemoglobin 9.5 g/dL), thrombocytopenia (platelet count 125 000/μL), and elevated liver enzymes (AST 75 U/L, ALT 80 U/L), prompting further investigation for an underlying systemic condition.

### Diagnostic workup

Given the clinical presentation and radiologic findings, the patient was referred to a geneticist for genetic testing. DNA sequencing confirmed a homozygous mutation in the GBA1 gene, confirming the diagnosis of Gaucher disease type 1. This diagnosis was consistent with the non-neuropathic form of Gaucher disease, which typically involves visceral and skeletal symptoms without significant neurological impairment.

### Surgical management

Due to the patient's functional disability from foot malformations, corrective surgery was necessary to enhance mobility and quality of life. Careful consideration of her health, including Gaucher disease, preceded the decision. Preoperative imaging revealed osteoporosis in the left foot, particularly in the metatarsal and phalangeal bones, necessitating a meticulous surgical approach to reduce fracture risks.

**Figure 1:**
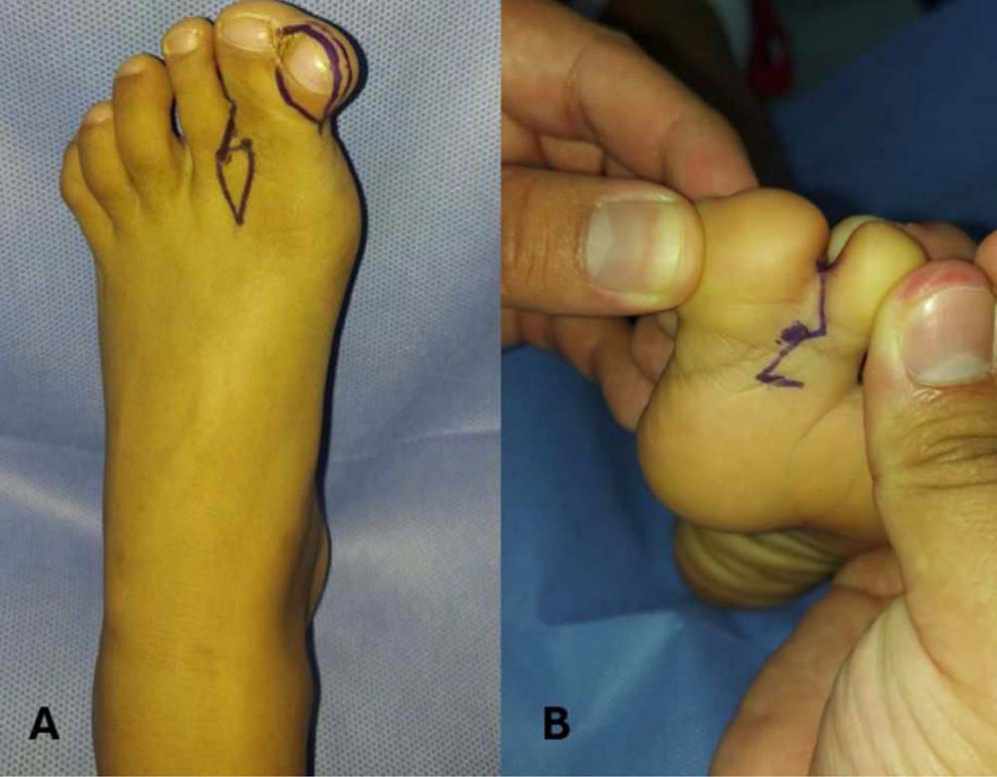
Preoperative marking in a patient with hallux duplication and syndactyly of the left foot. (A) Dorsal view of the left foot showing hallux duplication and syndactyly between the first and second toes. Preoperative markings using dermographic ink outlined the resection area for the supernumerary hallux and the design of a diamond- or rhomboid-shaped flap to aid in interdigital space reconstruction and optimize tissue adaptation. (B) Plantar view of the same case showing syndactyly between the first and second toes. A zigzag marking pattern was applied to the interdigital fusion site to reduce the formation of linear contractile scars and facilitate proper digit separation after surgery.

The surgical approach involved two main objectives: excision of the accessory hallux (the duplicate big toe) and separation of the fused toes (syndactyly) to restore functional anatomy. The team planned for a staged procedure, beginning with the excision of the accessory hallux. Given the patient’s osteoporotic bone condition, it was crucial to avoid undue stress on the surrounding bone structures during this part of the procedure (Fig. 1). The accessory hallux was removed using precise osteotomy techniques to minimize damage to the adjacent tissues and bone, which were brittle. Careful attention was paid to the soft tissues and tendons surrounding the toes to preserve function.

**Figure 2:**
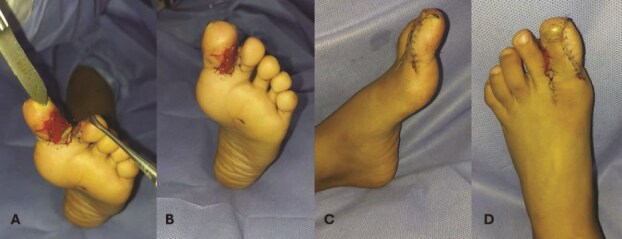
Intraoperative phases of hallux duplication and syndactyly correction of the left foot. (A) The surgical phase shows the complete removal of the supernumerary bone fragment of the left hallux and excision of the nail plate to ensure proper soft tissue approximation. Integra, an artificial dermal graft, was implanted between the first and second toes to promote tissue regeneration and reduce postoperative adhesions. (B) After the structural correction, the anatomical separation of the first and second toes was achieved, with adequate tissue coverage using the pre-designed flap. (C, D) Primary wound closure was performed with U-shaped sutures (6–0 nylon), providing stability in high-tension areas and ensuring proper alignment of the wound edges. The integra graft adhered well, supporting optimal wound healing and reducing the risk of complications like dehiscence or infection.

For the syndactyly correction, the fused toes were separated carefully. The surgical team made an incision along the fusion line and meticulously dissected the soft tissues to separate the toes, ensuring that the blood supply to each toe remained intact. The toes were then realigned and secured in place using absorbable sutures to promote optimal healing. Postoperatively, a cast was applied to immobilize the foot and protect the surgical site while healing occurred (Fig. 2). The patient was advised to avoid any weight-bearing activity for a specified period to facilitate proper healing.

Preoperative planning addressed the patient’s splenomegaly and thrombocytopenia due to Gaucher disease, with platelet transfusion and hematologic therapy to reduce bleeding risk. Intraoperative coagulation was monitored, and hemostasis ensured proper bleeding control. Elevated liver enzymes were also tracked for potential complications. During surgery, the team used intraoperative X-rays to check for fractures due to low bone density, with no fractures occurring. To support soft tissue healing after syndactyly correction, Integra Dermal Regeneration Template was applied to the surgical site. The template was left in place for 14 days, followed by removal of the silicone layer. Complete wound healing was achieved by day 28, with no complications observed.

Postoperatively, recovery was uneventful, though the patient experienced persistent pain and weakness, leading to the initiation of physical therapy to improve mobility and strength.

## Discussion

The surgical management of this pediatric patient with hallux duplication and syndactyly, complicated by Gaucher disease, required a meticulous approach due to the patient's osteoporotic bones and thrombocytopenia. The surgical procedure was divided into two main components: excision of the accessory hallux and syndactyly correction.

The accessory hallux was excised using a controlled V-shaped osteotomy at the base of the accessory toe, minimizing stress on the surrounding osteoporotic bone [5]. A high-precision oscillating saw was used, and gentle retraction was applied to protect tendons and neurovascular structures. Soft tissue dissection was carried out to preserve blood vessels and nerves, considering the patient's thrombocytopenia [6].

The syndactyly was corrected through a staged separation of the fused toes. The incision was placed along the natural skin folds, and fine dissection preserved the digital arteries and veins. The toes were realigned and fixed with absorbable sutures, ensuring proper circulation and soft tissue healing. Due to the patient's low bone density, K-wires were not necessary, and soft tissue realignment was sufficient [7, 8].

Frequent intraoperative fluoroscopy ensured no fractures occurred, while platelet transfusion and coagulation monitoring stabilized the patient's bleeding risk [9].

Post-surgery, the foot was immobilized in a cast, and physical therapy focused on joint mobility, muscle strength, and functional capacity to address persistent pain and weakness due to Gaucher disease.

This case emphasizes the importance of a careful surgical approach, considering the patient’s underlying conditions, to ensure successful functional outcomes and minimize complications.

## Conclusion

This case report focuses on the surgical management of a pediatric patient with the rare coexistence of Gaucher disease and congenital foot malformations, including hallux duplication and syndactyly. The patient's severe Gaucher manifestations, such as splenomegaly and osteoporosis, complicated the surgical approach to correct the foot deformities. The need for a multidisciplinary approach highlights the challenges in managing these rare, genetically distinct conditions together. The case emphasizes the importance of careful surgical planning and raises questions about potential genetic links between these disorders.
